# Supplementary material for: Lepidopteran wing scales contain abundant cross-linked film-forming histidine-rich cuticular proteins
Source: Commun Biol. 2021 Apr 22;4:491. doi: 10.1038/s42003-021-01996-4 (PMC8062583; doi:10.1038/s42003-021-01996-4)
Supplement: Supplementary file 2 — Supplementary Information [file 42003_2021_1996_MOESM2_ESM.pdf]

Lepidopteran wing scales contain abundant cross-linked film-forming histidine-rich cuticular proteins

Jianqiu Liu[1, 2], Zhiwei Chen[1], Yingdan Xiao[1, 2], Tsunaki Asano[3], Shenglong Li[1], Li Peng[4], Enxiang Chen[1, 2], Jiwei Zhang[1], Wanshun Li[1, 2], Yan Zhang[1, 2], Xiaoling Tong[2], Keiko Kadono-Okuda[5], Ping Zhao[2], Ningjia He[1], Kallare P. Arunkumar[1, 2, 6], Karumathil P. Gopinathan[7], Qingyou Xia[2], Judith H. Willis [8], Marian R. Goldsmith[1, 2, 9]*, Kazuei Mita[1, 2]*

[1] State Key Laboratory of Silkworm Genome Biology, Southwest University, Chongqing, 400716, China

[2] Biological Science Research Center, Southwest University, Chongqing, 400715, China.

[3] Department of Biological Sciences, Tokyo Metropolitan University, Minamiosawa, Hachioji, Tokyo 192-0397, Japan

[4] Shanghai Center for Plant Stress Biology and Center of Excellence in Molecular Plant Sciences, Chinese Academy of Sciences, Shanghai 200032, China.

[5] Institute of Agrobiological Sciences, National Agriculture and Food Research Organization (NARO), Tsukuba 305-8634, Japan

[6] Central Muga Eri Research and Training Institute, (CMER&TI), Central Silk Board, Lahdoigarh, Jorhat 785700, India

[7] Indian Institute of Science, Bangalore, India

[8] Department of Cellular Biology, University of Georgia, Athens, GA, 30605 USA

[9] University of Rhode Island, Kingston, RI, 02881 USA

*Correspondence authors:

Kazuei Mita

State Key Laboratory of Silkworm Genome Biology, Southwest University, Chongqing 400716, China. E-mail: mitakazuei@gmail.com.

Marian R. Goldsmith

Department of Biological Sciences, University of Rhode Island, Kingston, Rhode Island 02881, USA. E-mail: mki101@uri.edu.

Supplementary Information

Supplementary Table1: Primers for RT-qPCR

Gene name	sequence	product(nt)
BmorCPR82	Q-BmorCPR82-f<br>5'GCGTACTCCGTTCACGACCATC3'<br>Q-BmorCPR82-r<br>5'AGCGACCGTCAGGTTCAACAAG 3'	109
	Q-BmorCPR152-f<br>5‘GCGACCACAAATCCCAGTCA3’<br>Q-BmorCPR152-r<br>5'CCGTGGTGGTCATCTGCATT3'	
BmorCPR57	Q-BmorCPR57-f<br>5‘GGCTCATATTCGCTCACGGA3’<br>Q-BmorCPR57-r<br>5‘AGCGACAGCGTGAGTTACAA3’	171
BmorCPR129	Q-BmorCPR129-f<br>5’TTCAACTACGGTGTCAAGGA3’<br>Q-BmorCPR129-r<br>5‘GTGAATAACTGCCTTTGACG3’	91
BmLac2A	Q-BmLac2A-f<br>5'CACGCTCACACTGGACTACA3'<br>Q-BmLac2A-r<br>5'TCTTTCAGCGGCATCTTCGT3'	159

Supplementary Tab2. All sequences of siRNA

**Gene name**	**sense（5'-3'）**	**antisense（5'-3'）**
siGFP	AUAGACGUUGUGGCUGUUGUA	CAACAGCCACAACGUCUAUUU
siBmorCPR82-1	CCUGACGGUCGCUUCAGGATT	UCCUGAAGCGACCGUCAGGTT
siBmorCPR82-2	CCAAUUCAUCAUGAUCACUTT	AGUGAUCAUGAUGAAUUGGTT
siBmorCPR152-1	CGCUGUCGUACAUAACAGATT	UCUGUUAUGUACGACAGCGTT
siBmorCPR152-2	CAAUCAUCAUCACCAUGUUTT	AACAUGGUGAUGAUGAUUGTT
siBmorCPR57-1	CUCACGCUGUCGCUUCUGUTT	ACAGAAGCGACAGCGUGAGTT
siBmorCPR57-2	CAAGGCACAGACAGAGUCUTT	AGACUCUGUCUGUGCCUUGTT
siBmorCPR129-1	GCUAUCCAAAGUACGCGUUTT	AACGCGUACUUUGGAUAGCTT
siBmorCPR129-2	CUUUGGUCAUUAACCACAUTT	AUGUGGUUAAUGACCAAAGTT
siBmLac2A-1	CCACCAAGGAUCUGUUACUTT	AGUAACAGAUCCUUGGUGGTT
siBmLac2A-2	GGUCACAACUUGACAGUUATT	UAACUGUCAAGUUGUGACCTT

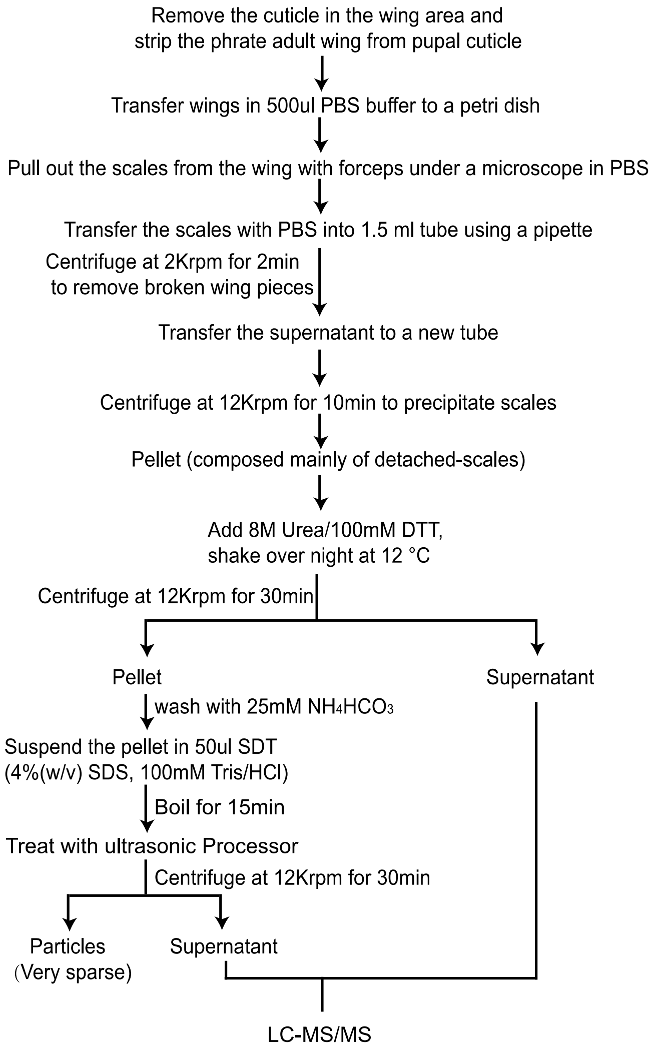

Supplementary Figure. 1 Protocol for collection and subsequent treatment of scales.

CPH

>BmorCPH3

MRFLIVSALLACVAAAPSHLVPFPAVAYHAVAIPAVVPTLSPGDIQAAAIDAQVKAADLAQ
AAADKAIAINDQNAENYNVKAVVNTNLAQEQAVDGVWAVEDKKWQALDALKTAEAQLD
GAVASQAVQLAKSAVGAAPYVVAPVFPVVYPGIASPAIKSIATQPPVEEVKTVADVEASA
KAVEGPAELEVGKVEGNTDSVAVEAKSATEAAESSAIQSAAKTSAVESDAQTSGVLGA
GHISTIQGAIATKTSYPTIPLVGPAFLAHPQVPLVFAVASPSW

>BmorCPH33

MIAFKTTVVLLLVALASARPSEEWEPEGHTHTEHTKPYHVTVVKKIGVPIPHPVAVSVPQ
YVKVPIPQPYPVHVTVEQPIHVPVYKVVHQVVEKPVPYTVEKPVPYEVEKPYPVEVEKK
VEVPIPKPYPVHVPVYKHIYHHKGGKH

>BmorCPH35

MSTVAEDSSTAGHYYVKLPQQRYLSVIKAEQRVRYTADDTGYHGAAALNASDDHHSHT
ANFALGQTAIDLNKLDYGGPTTTIQPANLVSSNTQPIDIFLIQQQFTSPSAQENNLQISSP
SNIQYSAPYITPFYQIQTQRPNVNQKNASKNPNEEDLIQEKTDETIVTPLPILQVFKNHKC
STENPNTDVASETVDDSLNSSKDLIADITSYEIHKINNFGLRDEAKRPLTYRGAVHFKVET
PRPNARNERFYYTTDYAPIISESNKVNITQEGIKKLVASTQDLISNEDLLKINHAAEKHVND
LSEDIIKPKPRFHAQTKSRNLKIENRPTEITVRAKIENILRDIENTSDKSKSNIIETNSNDYKF
ASPIVVPDNTYADFKEQIVNNLVSTMVPYIADGYQIVDVKSGYNNTSDIDISHDTDENVVD
VTPRPIGQNYLAPITVALRLLNANQSVTLNAVDDHEASDSEQISETVQSPKRERTIVEVQ
ESLPVEITHINDVEVHEYLEEGKSNDKEHLELAKSLYNTYIDALRSSKRIQDNSNKMLYQY
GTMNSYESSDSTEKQDYDSKENLEQSENMQSEVQVRPDEDNDRSESIDYYNNYENQK
IIQPIIVEKEVPVTKFVDRYIETRVPYPEAVPVPVDRPVPVAVPYEKIVEKPVEVTRYVDKP
YPVEVPRPYPVQVKVPYPVEQKVYVDRPVHVPYSVEKVIEKQILHPVPIPTPVGIPYAIQI
PVEHKILYPITVEKPVPIPVEVEKPVPVEKVVHKEIPVPYPVEKRVPYPVPYETKVAIPVPI
EHRVPVEKIVEKPVTVTKYVEKPVHIEVPRPVAIPVHIPQPYPVDRIVEKKVPYPVPVDRIV
EKKIPVKVPYAVEKYVEKIVEKPVVLTKYVDKPYPVEKRVPYPVEKIVEKRVPYAVQVPY
EVRVPYPVEKPVHIPAYRYYSDEEARHIIHYQKPDQRNTQTQLSQAQAYARYLQSLSDK
YRNNRNVQSTLWGNQYASSYQYFNKTDKQSGLKNAQNVANYIKYLQHTQSKSLNHNG
LNRNRSDFVGYQLRGAVLRAPKTLKIEYGGFKPPLVPSTEVDLDGIPVNKRDD

>BmorCPH36

MYAKIALISLVVAVVVASPTPGGGGGHKHVTIHVPYKIHTIHHHHVSKVHVPVHVPVIKEV
QVIKEVPIIKHVPVPIVKHVPFPVVKHVEVEKKVIVPVHHHEHHEHHEEEQHGWEGEALH
GWN

>BmorCPH43

MKAFIAVLFVVASVLGDPEPSKTIQKRSGHLGHYADLYGHGLSSGIAVHAGPAFGAHGLA
GPAIAGHAIAGHAIAGPAIAGPAIAGPAIAGPYGAGVIAAEGAGGAIIGANVHTTITRHVGV
PVPAPYPVAVDRPVPYPVRVGVPYPVDRPYAVPVPRPYPVAVEKHVAVPVDRPVPVPVP
HAVPVPVVKQVGVPVPAPYPVAVPRPVAVPVPAPVAVPYVKSVIGAHAHLCSAIGHDIH

>BmorCPH5

MSISKWLACALLLVCVSLRQSIAAPALQEEQYPPMPYEYKYDVEDPEKTLYFGANEVGD
AEGKVVGGYRVLLPDGRLMTVEYTVQGDSGFVPKISFDSNANPFGQGK

>BmorCPH28

MAAKFYVLCVLLATGIAAGARPRPRLQQVEDSYDYENQAQDSRAGGQVYQLVPADQYD
SIYRQNDRQDEEYEQRPVQRLPSRQKLQPQPQQEGPKQPPVQTIRNYNKVNDDGSFT
FGYEAADGSFKEETRGTDCVVRGKYGYIDPDGNKREFTYVSGNPCDPNKPNEEDEPE
APAPDSAERDDGVPNYPTRVAPRPTTPRPPTTFFQNDFRDADEDEDDEPVQAIQPVRQ
RVVQRPALRRPAYQPQPIAITPRPLPVTTARALPPATTFRPQLLQITPKPQIQYSPEPHYS
PSPSPIATTARPGPGQIDFAAEFAKFHRENQLQGSTSATAASPTKATAASPSPSGNPLYS
TELVYDPSSGQYNTQLFQTLPQTKGELNLNLRLQPFVAQQQSQPRPFVPSPQIPAVPSS
PASSAPLYRQQLQNPQEVYQRQQAEQQFQNSQQLFAQQQLQQSQLQRDRAAARAQ
AQRLAAQGQAAPAPQRASPAPQYYYVAPRGGESVSSGQIDAFLRGHGIQF

>BmorCPH30

MQSLVILAATLCLAQASYYLGAPAPIQLSPDGKYVLDTPEVAHAKAAHLAAHAQASTSHG
AWSPGYGGYASDAHYGAPAAGLYKYGPAPLAHDGRVIDTPEVAHLKAAHNAAHAAAHA
NAAHGALAHAGSYAPLAYASAPLAHGAGYAAGYGKWTGPQAHIQLTHDGQYVVDTPEV
QHARASHLAQYHAAAHAAAAAPEESWAPHGHGWH

>BmorCPH31

MKSMIVVACLALACGAHASGWAGPPANIALSQDGRNILDTPEVAQARAAHISALQQASK

NNPNPNDDGSYDPRWDNEEYWQQAEGKWNGAPAPAWNAAPAPSWNGAHAAAPSW
NAAPAHSWNAAGSAPAPVAETPEVAQARAAHLAALSAAKSAAPAQQQWNAPAHQDWN
APAHQDWNAPAHQDWNAPAHQSWNGAPSWQSGAPAHQPANIRLANDGSGILDTPEVA
AARAAHLAAHAQAAHSAPAHAPQQHW

>BmorCPH41

MSLVSCAVSEEANKINSDNVKKYQHELLPKLKDLTERVQLLSWALQQNVIDTYINYTVTK
SLEQLNFKSRKLKIDNDYNTLEEELRTFTEDFNDNDGIMKNAEALHKSYHILKDLCVLGE
AKSVINRANHEFNRYNYSEAMLSLKNLKGQLDNVKADGNVAKVVLNLYDHVENQLALFT
AHLSIEWEDVFTWSEKKGLNFLIYSLSVQQSDQVLLQRILNTLYVTDRMKAELWSFSDFF
INKLVHNVIRHNCDIFTEDHIGAIVFNIKIDLSDNTKPNYQTIFNNLTAIFEFLQSTLGSQFQS
DETFTQIFAKSIKSDFFDKIIEDCVRSNLQSYDGSYQSYKNIVIELDSFNKFLIEIKFVDPDD
SPLNSYIENTECLLYNKKCEKLLINVRSLISQSLSYDSIEVGSTLNDNKTDQSNNEIPWDL
NAPVYLPKCMISRNVKTIMTLIKDHLEESSKLPEKYSMKFVSYIKDIAVMYQCVVPRKFKI
NLESCPSDIGMNDNYPLIKVLIHRILLTSMVNFFTALFFNNCYYLAHGLVGPPWCTTLPQD
LADRLVLVLFECIQGLRVLGLEKLSLYLQQQKTVIQEGIQPKESTSWTHEEFEHFDCGLN
RAMTLMKELKTSWLHILPSRMYVMSMCTLIEVLCETVLNRIFCDIKHVSEDLVYLIATRIED
TLEEVVTLFEEPIELEEQISIWIKFSRMPLLLKAQLLEISELWNKDRELFSDYTCEEFRHVT
ALVITVLVIVVHQGNGVAAYDDGRVDQIAEEAKNRAVTIIDGPKYVEPPRSVDVLLNTSNP
APNTNNNNSNVNVQHPNNTKPEQRPPSKPPITPDKPGKQIYFPKMPRPTYYAHIQKIPQ
PRHHHQPPFKYYHSPNSIYKFPRVIPLPPHLRNHYYLKSQPLMFAASTGKPSIVKSPKFS
LPVQTINGIRTEFVRPPKFWNITSTTTTTTTTEATTTTKALRTKRIWPKRTSELDNSTLSN
ATVLDNSSLSNSSNEDYLETSATFRRFRYAYKNATTTSTTQSPTLRSYRPVTRIPKIKSTT
PTINFTAIGPNDWVPIVPSHFPKLRTLLAPIPTPINKRSDFITNSETPPEKQMMYLQSFGLV
PVSKNSETAMTRKKMVFFKRKRQLNPYYSGYSPRPGKPLKGVIYGDQETDESHSHPRV
VTKIKHHHHHHHHRYIKTVEKPVKVPYKVEIPKPYPVTVEKKVPYPVEKIKFVDKPVPYPV
TVEKRIPYPIGIKVPHPVPVKVVEKEYVPKPYPIVHHVPVVKHVQVKVPHPVPVPVEKKV
PYPVEVKVPVDRPVPVRVTVEKKVPYPVPVKVLVPQPYPVETKVPYPVEVKVKEPVEVI
RHVPVKVPVPQPYPVKVPVRVTVEKKVPYPVEVEKKIPVPVEVYIPQKVEVEKKIPVYVP
KPYPVEKKVPVPVKVPYPVKVPVQVPIEVPIYIHHPYQIESADYISNVQQYDPQNVGQYF
SNIASQFFSNTNKGEQRIFGTISAEVSTNSPQHTVTAHGNSGFAGFHSRSGTDTLTTTAA

TTI

CPG

>BmorCPG3

MKSFVVICALVALASAGNVREKRGFLGGLGGYGGSFGGGYGGYSGLGGYSSYSAPSV
VSVNKVVNVPRVVSVPQVVHVNKVVAQPQVVTVNKVVGGIGGFGGGYGGGYSGGYG
GGYGGGYGGGYGGGYGGSGWW

>BmorCPG9

MKYHIFLGLIAAVTAFPGIIHDEAPQVEAKYDHQSLIGESGHHHQIEHEHAKSHQSIKFEH
FHPVPVYVKKEHSHLLKHPLEKGKSEQNLKLIHPETEHKHGGGLVLEDDRHNIEHHIASA
GLEHGSFEHGGLEQGLEHGGLEHGGYEHGGFEQGGYEHLSGGFKGYEGGEGLKTYA
ELQQPEDHGHYYAEHGQALSSDSGEGYKYESY

>BmorCPG20

MKAFVACIALALVSCAYAEAPGGYNYNRPSGGGGFSGGFSGGLSGGGSYQAVSSGYQ
TSEGQNVDPQLLEQIRQILLKEEASSSSHGGSAPSSSYGAPSSSYGVPSTSYGVPSARV
VGLQLEGVRQAIQVAQYEQSGHGSGGSGGYPGRVPSGSYGAPY

>BmorCPG31

MSRLTILFVIAVAYEALGDGAVKDSRAEQSGYIGHSLAGLGSLGVGGAYIGGPAAIGVAAP
AVVAAAPALGSGAALNNAAAVGAAQLSAIQNAQAVQAAHIANSAAAAIQGARVTEAAARA
GQAQAITNARALDAARVANIQRAQAAAWENARAAEAARRAAAASAAEVARAVEAERLA
NAARVQAVAIANTRAVEAARIANAARAQAAAVATSAAQAQAVADAVARNAGALEGAVLVG
GGGLVAAGGLGSGLAYGIGHGYGAGYGYGAGYGLGHGYTVGHGYGGGKYIH

>BmorCPG35

MFFVNLILDSLQILVSYFVIQCYFITIGKPLFKGISIKKGFDISFIPLPKKPLSFITEKWSPFGI
FGVKHAIQKRELIEEEEPIVTMKTSLQPPPMPTISPLPPTSTGPMRTILINEDVLRLPIDDSI
ETMSSAVFPVPLEPPLPSNFVIIPTTKEEKGPSGFYTDISRAVPVNNYLNLVERPAFPAVA
PVVENELPPNTPSVPKENPNVDPLLENNSIVRGSKLALYFSNLFLQLFSQFLANARVTLN
QFSEPTV

>BmorCPG38

MTPIYVLMVLFVASSLSRAKEIKEVHEKAVKRHVPLGYGYSHGLSGGLVGSGIGYAAGHS

IGVGAPAIGIGNGPVGFATSGIGSGIGIGSGIGIGSGIGIGGGAGIGFGGGSAGIGGSGVGI
AAGPAIGIATGPAVGAVGIAAPSVSVGAAPAVSTSVAVEPNPVILRQEVPRYITRTITRDVQ
VPVQVPVPFPVDRQVPFPVRVPVPVQVDRPVPVPVVHRVPVEVTRQVPVYYEKKVPYP
VKVPVSVPVPYPVTVEKHVPVDRPVYVDRQVPVPHPVYVDRPVNVPVPVQVDRPVPVP
HPVVVERRVPVPVSVQSVAAVPAVVGVSSIGGAGGIGAGGIGAGGIGIGGVGIGIGSIGG
GGVGIGGLSGSGGLGIGGLSGYSGVSGLSSYGGYASGVPVVSSGFSSIPVSGHVSLSH
GIPLSGASSYSKYYSHGH

>BmorCPG39

MVRINYIFLVFCFNYALSAEQKKRSYVGGGSHGFSSGGAISSIGGAGIASSGIISSIGSGIG
AEGVGGGFVSGGAGGIGGIGGFGGIGAGGVGGGSIIGSGSLSGVPTQHTVSEYYNTVA
VPVPQPVPVSVPRPVPYPVHVGVPVDSPRPVPVPVPQPVPHVVTRPVAVPVDRPYAYP
VAQAVPVTVTQSVGIPVPQPYPVSVPAPVPVSVPQAVSVPVTIVASGSGGGLGLGGGV
SGFGGGFSGIGGGVSAVGGGIGGYGGTVGGFVPSSGIGLGVGGGIGHGIGSVTLGSSL
GSSLTSGSLSGHTIATSHGHRIHHSY

>BmorCPG41

MNSFITLTCLLAVVGLSCGSAIGLGYGYGYGAPILAPAISTANVVKAAPIPIIRTAVAPIITAPII
KTVAPVSIGYGLGSYGWGGHSLGGYGWGGHSLGGYGLGGYGLGGHGLGGYGLARAY
GGWGSGLGNWGWSKH

RR-1

>BmorCPR10

MYRLIVSAFVIVAVSCQQHEGARRVPKYAGDPKTAAIVQEARYLSGNGAFGAAYQQED
GINFKEETDAEGNRKGSYSYIDPSGQRKTVNYIAGKNGFQAVGDHIPTAPQAAAPTPGY
TPDPRYNSPDYKAPQYSAPQQYVSQAQPRNYGGKPNEDDGQYYPELYEQENYAAPQ
PRYQPQQQYQAQPQYQAQPQPQYQPQPQYQPEPQPRYQSQPQYQSTNIYTGVQQA
QYSGLQTQQEYSAPETRSQYYEPTTPPPARFFPPGKFSLNRAPDGYSYSFHKA

>BmorCPR33

MISILLCTLAVFYAVHAQQHSINDPIPIIRYESDGPNPDGSYKWLYETGNEINAEETGYVK
NFGKGEGEEVQVAEGKFSYKAPDGSLIALSYIADENGFQPQGDHLPTPPPIPPAIQKALD
YLKTLPPSAQDSSNSQGQYQQPAPFKPRGRF

>BmorCPR37

MTMKSIFLFGLVAAALAAPQPQKSDQTAEIIKQDFDQQVDGSYQFSYETDNGIKAEETG
SLKKASGPDASDVIIAQGAFSYTAPDGTVISLNYVADDDGGFKPEGAHLPTPPPIPPAIQK
ALDFLATAPPPPSSRN

>BmorCPR41

MRVFLAICLSLTVALAAETGKYTPFQYNRVYSTVSPFVYKPGRYVADPGRYDPSRDNSG
RYIPDNSGAYNGDRGDRGAAGGFYTGSGTAGGPGGAYVGTKEDLSKYLGDAYKGSSI
VPLPVVKPTIPVPVTPTYVASKVVTPTYVASKVVPPSGAGYDYKYGIIRYDNDVAPEGYH
YLYETENKILAEEAGKVENVGTENEGIKVKGFYEYVGPDGVTYRVDYTADENGFVADGA
HIPK

>BmorCPR52

MNYLIIFALAAVAADRLDKDLYLPPHAGSSGGASAGLQGPRNSAGSHQALTGSQSGQP
AEILRYDNEINEDGYHYAFETSDGTKAEQEGQVVPGAKPEEGSINVKGSFSYVGDDGQ
TYSVSYTADENGFRPEGAHLPTAPPIPEEILKSLQLTD TKRDQYSSQKSSYDADAGY

>BmorCPR15

MKLFVVAAVLGICLADRLDNKYLPPRGNAGAGFGPGFGLAPGGGGFGGNGGLGQGGF
APQPSGGYNAASQAYSSRSQASADAGAQILRLNNEVTAEGFSYDFETSNGIRADAQGV
ATNGVQSQGSFAYKGDDGQDYSITYTADENGYQPQGAHLPTPPPIPEEILKSLEQNARD
EAAGIVDDGTYHGEGAGSGAAAYSSSGSAGYSSGGHSSGFGGAGSGAFGAGSGSGA
GAGGFGASAGGFGSRGAGFGGAASRQYLAPNAGSRGSGSGNFNAQTGYKY

>BmorCPR34

MKLLIAISALIAVAAALPQRKVSLKPEQQVEDQLPLEQNFNNYQPQQQEYRSAKPVDDF
RPKVQLETSTYIPIIRFDKEQGTDGSYKTSYETGNNIQAQEQGYLKTVGDNQDNTALVQ
QGSYTYTAPDGQVITVEYTADEFGFRVSGDHIPTPPPVSAEIQKGLDLIYAGIKANQERA
AIEAKSNPEAARQQEEKAALDYKGQYYQQ

>BmorCPR48

MRLICFWCLFVTTVAAQADFYEPQTFKQNLDRYQKSLFTPTEDVTPQGSYMQAWRPGP
SDHNTVASVPAPILPKPEPWKAPYPSKEKQAAILHHKQALTSEGSFRFEYASDNGLAAG
EVIEPDGSRVGAYQYKDPNGQVVKLKYRAGKEGFQILEGSHLPKSPEPVAPHTPDNYY
QQAYAQQREQYLQQQYNQQRPEPQNSWRQDQGYEGSQRPGGGQYLAQNWRPQD

LNEDDGQYRDNEVEQRGPHSFGEGYAFAFKG

>BmorCPR54

MTPFARLAFLGLIACAVAQYNEDRAPRYIATEPKEVSTPVPILKQINRHNEDGSYTYGYEA
ADGSFKIETKSQAGDVKGKYGYKDDTGKLRVIEYGANKYGFQPAGEGITVAPPTLVDES
TRDEGLRPGKSQGGRSQYRAPAPQPDYDYEEPAPAPPPRPRPQPQPQPQYRAPAPRP
QPQPQQQYRPAPQPQQQYRPNPQPQQQYRPEQSAAPTPPKPAFFSGAAPAPVENNFF
EPEPAPRPRPKQDFRPAPQFQSFAPAQDFAPSPPQQQRYTTANQFPQQKSQPYSMLD
QLLKEYSLPQGGAAPLHDITFGSY

>BmorCPR56

MLCHTLILVLAAGLTSCDVSHLETTTPDPPPKPYVFSYTAGRFPGHVDREHTEVSDGSG
VVRGKFAYVDPRHKVRTVDYVADKEGFHPILSDVPPEHPADSESVALAKDRHFQLYSKIA
EEHAQHPHPYETSVPRQSAAVAEATLKHSELFRVIAEQHARIAAEREALIREEEEKQHLQ
ELEQ

RR-2

>BmorCPR57

MFQYIAIACLLGVAAAAPSATPTAAAYVAAVDSVSVPHYGFNYAVNDPHTGDNKAQTESR
DGDVVKGSYSLTEPDGTIRVVDYTADSVTGFNANVKRLGPAAHPQTLITKQAIVAPVVTH
AVASVAHVPTSLISVGIGHGHGGHHYAGLGHIGHGHGGHDYAGLGHIGHYGIGHVGHAD
LAHIGHGHIGHLGLGHVDLGYAGIGHHAHVGLDHIGHIGLGHGGLDHIGHIGLSHGGLDH
ISHIGLSHAGLDHIGHGLSLKH

>BmorCPR68

MALKLVTFSCLLAAAYGSVVPAAYAAAPLVAAAPAVAARIEEFDPLPQYRFGYDVADSLT
GDYKSQQEERNGDLVQGSYSLVEPDGTRRVVDYAADSINGFNAVVRKEPLVAAAPAVV
AEPAVVPARYAAAPVAAAPVAAAPIAAAPVAAPYYA
ARYAVAPAYSAPYVTAARYEAAPFVARYAAAPVAAAPVAAAPAPIYARYATAPVAPAPV
VAARYAAPSVVAARYAAAGPYAAYTAPFSAAYTAPFSAAYTSYASPVAAAYTTYTATGP
VSAAPVAAAYAAPIAATAVPAVASTVRAAPAKLIETTA AGYRYP

>BmorCPR69

MDSKIVVFICLVGVASASVIAPVPVARVDPLPQYSYGYDVQDTLTGDFKGHQENRNGDL
VTGSYSVVDPDGTRRIVDYTADPLNGFNAVVRREPLVVAAPARVVAPAPIVAPAPVVAP
ARVFASAPIVAPGPYYARAPFAPAPLLAPRLPAPVYF

>BmorCPR70

MAFKVVVFSCLVAMACASAPLVAEPVVAAPAVAARLEEFDPLPQYRFGYDVADSLTGD
YKSQQEERNGDLVQGSYSLVEPDGTRRTVDYSADSVNGFNAVVRKEPLVAAAPAVVA
EPAVVPARIAAARVVAAPVAAPVVPVAARLAAAPVVAAPVAKYTAAYTAPLAYSAPVAAA
YPAPLRAAYPAPIARYVAL

>BmorCPR71

MAFKLAVLVCAFATVRAGFVPAAYTAHPAPVAYAAAPVVHAAPAAHLIHASPVAYAAPF
AKVAAVAPVAKVEEYDAHPQYSFAYDVQDSLTGDSKTQHETRDGDVVQGSYSVVDPD
GTKRTVDYTADPHNGFNAVVHKEPLAHVAKVAKIAAPLTYAAGPLVHAAPVVHAAPIVH
AAPVAYSAPIYHH

>BmorCPR73

MKVLILAAFLAVCRGAAGGLAVPAPYSPYAYARPLGLAAPLAVARPALASYAVAPAIAKVAA
PVEEYDPNPQYSYAYDIQDAITGDSKSQQESRSGDVVQGSYSLVEPDGTRRIVEYTADP
HNGFNAVVHKEPIGGVVAKAVAPAYLH

>BmorCPR74

MIAKIFFLCCACASVIAAPGLFDGYGYAAPAYAAPVAHAVAPAYAAPIAHAVAAPVVKTVAA
VHAEPIDPNPAYSFSYGVQDPATGDHKDATETLQNGVVHGSYSLVEPDGHLRKVTYTAD
KINGFNAVVERTGGSHAVAAAPVAKVVAAPVAIQAAHIGHVAHPWG

>BmorCPR78

MLRKCVTLAILGCVLAAPQHQYQAQPQQHGEGIPPHLLRQYLADQGGAQTHTISRPTHI
APAPARIPYAVQSEPQYQQQAQYQPQPQPQPQAQPQPQYRQQYQPQRAEPQQPQEE
YDPHPSYQFGFDVNDDQYTNYQNRKEQRDGDVIKGSYSVVDSDGFIRTVTYTADPKEG
FKAEVQRQPTDIVVKIPTPKPQIAQQPQLAHQPQISHQPQIGHQQQIGHQSRPQQPPPQ
YYHYEQ

>BmorCPR79

MKAISFTMVLILANALAYHDPDLNYHLSQVQKVSNCADSGYSYPAPGIQLTHSGINVAPA

PAVVSQPTASIKYTPAIQYAAQPTYQTVESNYITAPVYTKELHGYATSAGLSSSASTTKTV
TPLATYAQAPIIAKITAAPLIAKFSVAPARTTYYSQNNVQQASYASGSSAKASLYSNTVHQ
GPVVSQVFAAPTVRYAASPALKAQQNAQVDSTQYSTGVQYNQVQNLPALQIPPGIQYAA
PSVSQVPVPRITQYFTPTQYSSGASYIASTAAQYLPTVKHAGAALNQVVVPISNGYSKGL
SSAPAYSSASFTRHSSPAVAAYSRPAIAPVSTHYSTPVISHYSTPGVAQVPEYLGARSNY
ASTPSLTIGGHISSANSVKNVHTEFLENYDAHPRYAFEYGVNDPHTGDIKQQKEERDGE
VVKGQYSLVEPDGSVRTVDYVADWETGFHADVRNSKDNQH

>BmorCPR81

MIAKVVILLSIAVCVLGGGHATSEQKVVVRHKKIINADKKSDEYAWSYPSYEFSYKVHDP
HTHDKKGQSENREDDEVKGEYWLIQPDGHKRIVSYHGDKKSGFNADVKYSEPHKHIDE
EKKSHHIPHYPEVEHKNIIVDYKKEEKEEGEKQEEEEEQDNKEEDKDGHSSVNLGEYEP
LPYRAPVVHKIRENYHHPHYYRKNRRS

>BmorCPR82

MYTTVTALFVLVSACQAIFPFHHHHPAISHQEIEKHVGPHHTVGIHHPVPIPIHHHVPLVHH
IPHHHIPIHHDHYAFPEYKFAYSVHDHHTGDVKSQHELRHGDVVRGGYELVEPDGRFRK
VEYKADDHTGFNAIVHHSSPHHHFHQEHHHI

>BmorCPR84

MFAKILTLAAMVAATQGGLLGHGLGHGDYSISSQHNLVHHDAHYAAAPLATLAHATPLA
YDGHYDAGHLSHGATLAHAAPLIHAAPVVHATPVVHAAPVAHLGAYSGHGHEDYYAHP
KYKYSYSVEDPHTGDHKSQHEVRDGDVVKGEYSLLQ
PDGSFRKVSYSADDHSGFNAVVHNSGPSHHVYSSQHHHY

>BmorCPR86

MFSKIVTFGAFLAAANAGLYGHGHAVSSQSIIRHDEAPLHYAHAAPVAHYAAPIAHYAAPI
AHYAAPVAHYAGPVAHYAGHDEYAHPKYDFAYSVADPHTGDHKSQHESRDGDSVHGSY
SLVQPDGSVRKVDYTADEHHGFNAVVHNSAPSVHPIAAHHHHHY

>BmorCPR87

MFSKIVTFGAFLAAANAGLYGHGHAVSSQSIIRHDEAPLHYAHAAPVAHYAAPIAHYAAPI
AHYAAPVAHYASPVAHYAGHDEYAHPKYDFAYSVADPHTGDHKSQHESRDGDSVHGSY
SLVQPDGSVRKVDYTADEHHGFNAVVHNSAPSVHPIAAHHHHY

>BmorCPR89

MFSKIVAFGALLAAANAGLYSHGHAVSSQSIIRHDEAPLHYAHAAPVAHYAAPIAHYAAPV
AHYATPVAHYAAPLGHYSGHDEYAHPKYDFAYSVADPHTGDHKSQHESRDGDSVHGSY
SLVQPDGSVRKVDYTADEHHGFNAVVHNSAPSVHPSAAHHHHY

>BmorCPR91

MIAKAAVILSVVALASAGVVQLAGHGYGGEEYGHEAIAYAPVAVAPVAHYAAHEDTHVDY
HAHPKYDYSYSVSDPHTGDHKTQHEARDGDVVKGEYSLLQPDGSFRKVTYTADDHNG
FNAVVHNTAPAHHEYHH

>BmorCPR125

MARDMILLILFANLLTIFCQEETAQGSTEDQQKDDGKLSYSFSYGVADARTGDVKAVWEA
KEGDTVKGQYSVLEADGSTRTVEYSAGPNSGFNAIVSNDNDFLPTNEIESKKTGRSLIE
DKTMRDYGNYYDFPEDPDDEYYEKKKTKRPLDSHREHSKNKKPRYPFDLEPSEYTHSI
SIKHPRDEGSESEAHSHFGYSFDPNCKTKPKKGSHDTNSYSNVVDLETNPKYPLYSQD
YFRDKHPDSSSNYDFEKLRPFSSYRPHKYEEITLKPPFSTRYTSPVIPDLAYSSEKMYPD
DIPLRPKKKHRPHKVPESHFGDDLDDYVLVPKKKYKPPRLVEPHEFRPEPEDDYERPHR
GSSFDDIHDDRHRHPPRGPQTEIVRKIVKKRRPVINLLDVFDI

>BmorCPR126

MRAFQVVIFVAVCGAASAGYIAPGYSGYSGYSGGLGGYNAGYSGGYGGYDGGNHGDY
YAYPKYAFDYSVNDPHTGDHKTQWESRDGDVVKGAYSLAEPDGTTRIVEYTADKHNGF
NAVVKRIGQAHHPQVYGGHYDGAGYYGYH

>BmorCPR128

MSLKGLILLATVACAHSRVLTYFRPTKHVDLQPSGTILHAEPHLGFEHHHISEDEPVDYYA
YPKYEFKYGVNDFHTGDIKTHYETRDGDVVKGQYTVVEPDGSIRTVDYTADKYNGFNAI
VHKTAPISPHEAAHLHH

>BmorCPR129

MQMLILLAFVGTSLAAPIDYFHGGVSYASPYAVHAPLTVHAAPVAVHAAPLVAHTPVAVHT
ETVSYPKYAFNYGVKDLHTGDIKSQQEQRDGDVVKGSYSLVEPDGTTRTVHYTADDHT
GFNAVVQKSGHAAHPVVAHVAPVAHVAAAPAYTIPHYGFH

>BmorCPR133

MELTSKTFALLLVVGSALAQYEDFGGFEGYHHKPISHQQHQASDEHHGQEYELDYHAH
PKYSFDYSVKDPHTGDEKEHWETRDGDKVKGTYTLVETDGTKRVVEYEADDKNGFNA

VVHKIGTPKHEEYHAKPAHEPVQYQHDYGHFDEGYAPISASFEHSHGFLAHKHGW

>BmorCPR135

MSFVVAVSCLLAVTALAKADDSWGGLVYAPRHASVDYYAYPRYAFEYSVNDPHTGDKKA

QWENRDGDVVKGAYSLVEPDGSVRIVEYYADAKSGFNAVVKRIGPNLHPPTVHAAPISPI

VGPVAKLGGLAAAPLITGPLYGGAVSTASLYKDHAPAVIPAPILPVSYKAPLPYAPAPIWPS

AAYPTPIIKSGPILSAPIYPAALPGLKTPFNLGDYGSLGLWNDGLLKAPLGPNNYLDHLGH

GLLTKGAYPSLGPNYSLLGLKH

>BmorCPR136

MNRLVALTVLHMATLATGIIIHNAPVVEAVDNSRYAFNYAVNDPQTGDKKAQWEERNGG

VVKGSYSLVEPDGSVRVVDYTADDVSGFNAIVKNIGPRVHPAPLTPKIPTPLAPIVGPTNY

GFGTEPVVGIPKATSIGHWSLPWDPKTHSYGGWAPITAPLLPIPLAPSRAYATILRKKYVD

GKLYKWITGPIPLSGKTLIIKQTGRGR

>BmorCPR137

MKTNVMIACLLVSATAVLANPPATLSLIPITTVDRISDPSYSFNYAVNDPSTGDNKAQWEH

RDGDQVRGAYSLVEPDGNVRHVEYSADPLTGFNAVVKKTGPNVHSLSIVAPIAPIAPIAPI

AHVEHVPIAPASHVAPVAEIAPLVLPHAPIAPLVHTPIITPVIETAPIIAPHPVNYNPLYRIRGP

APWVTLSGSSYTKGNVVRRWTAGPISLEGKTLTIKTRHH

>BmorCPR150

MFGKVVVLCVLVAVARAGLIAPHGAVSSQSIVLGTPAIAHGYAAPAIAAPAYGYGLGHSAV

VAHAAPVLRAPLASLAHAAAPVEIYSHPRYQFNYGVTDGHTGDQKSQWEARDGDVVK

GQYSLVEPDGTVRTVDYSADDHNGFNAVVSRHGHASHPAAHVVAAPAHGHYLG

>BmorCPR152

MWFQVATLCLVVIAAMAHPPATSHQFRVDHHDHHDHHHHVDHHNNHHHHVDHDHHSH

HDHHRHFNHNDHHDHHDHHEHQAYHGHHGHDGHHDHHGHHGHHDHHVTHQQHGH

LDHASSYPSYQFSYSVDDHHTGDHKSQSETRHGDHVTGEYSLVEPDGNVRSVHYNAD

DHHGFNAVVHNRFAHHHIIPHHDHHHHHDHHNHHDHHEHHDHHDHHVHHDHHDHHD

HHDHGHHEHHSHHDGHHEHHHDHHH

Other

>BmorCPAP1-F

MKRFLLGGFLFLSVLVEIVQPTCVLESDFGFKINCAFKKSGLFRVRNLGGIKAHASLGFSL
GDELGFEQSLTNLDPSRRRSVNIKNGAPPNLVADTSRLNAKQEKRAKQNRIMMRPVAPP
LTRPDQAAMDKLTSLHRRVSTMQPIVVPNPAVETPTNVPTQTMAKPMPMGVPPFRPLP
PKEDTLKVLPITSSTRKNYAPLQIPESKGPGLAYQSEIYPQSHNQISKMVSQMTAVQYAP
VVKIKPTPVILDPFHGVSTLKSINPFVPMPAPTQALPIIPSPATHAQSVHKTNPVSTFVDPV
PHHVHNIASSYVYNTKKVDDYNTITGYGDDTVLKFNKEDINAKIAEIAKVGNISMEAVEAAI
ALRQQQLLNKYAHLPAPPTTSSTTTTTQPPVVFQPEPEILTAALPQKPQSTRQPTSGKVM
NAPREYYPVGYEKNFDDHFQSKVDLPDTSFHCGDQKYFPGLYGDESLGCMVFHVCALT
DDGLVMKSFLCPESTLFDQTILKCNWWFYVDCKNTRKLYDTNIPVSKSYQLMKALTFFS
SYKKDNNMQDDGRSPNPEDVDGIKEAISILENQDTTKTAQTETGSDGLHIITPQPLIDERS
NRATPPAPVYRGDRQLGNTTRSGQNSTQPHSQTVADSSQEPRPFSAETDRDKKHRRR
MTLRFSNSTTSTKAPSVSTTHAVEVIQQEIQPIENLSPVRRETLADQLGTDARVEEAPPV
KRVYRSSAEKYTKEPTVAKHEELLIVTPTTFRLVGEATQKAAIESLDRAVLGGDGWGAAL
GGRHVAPAPRSPHSVIAYLVNLFIKIRVACLLNFFFLINSPRTFCDFQTKQAHLTFA

>BmorCPAP1-A

MCSAHGSNIVLRYEPYGLQFEQALIRKPLREHEKPQDLRNVPGTPGVDYPIYHSVPETR
FSCEHVPIHPGMYANVETGCQAYHVCHDGREGHQGASFLCTNGTLFDQTKFACDWWY
NVDCSQAIEHYKLNADPLKNPYVPKQKPEIHQEQPEEYNIPEEAYFRKY

>BmorCPAP3-D1

MSAKFLYLAACVAFANAGILLEHAPPCPEEYGVQAYAHPEQCDQFFLCTNGTLTVETCE
NGLLFDGKGAVHNHCNYNWAVDCGHRTANLEPLSTPGCEYQFGIYPDSHECSTSYIKC
AYGVPEQFPCTPGLVYDERSHSCNWPDLLQPFCNPEAVVGFKCPSKVPANTPSAKFWP
FPRFPVPGDCHRLITCVEGQPRLITCEEGKVFDDQNLTCEDPEIVPHCGRA

>BmorCPAP3-A2

MKSLIVLGLAVCGLVSGQEFKCPDKSGFYPDPYQCDLYYKCSRGDAEEKLCPDGLVFSD
ENPNKEHCDIPSNVDCGDRKELQEPKPSKGCPRQNGYFKHPDPQACDKFHYCADGIP
NELPCPPGLYFDEETSNCDWKEVVNRQCDQITKDVLDDGFTCPDGEVMGPNGRSLPH
PTFPHPEDCQKFYICRNGVQPQKGSCPSGKVYNEDTFMCDDPEKVVGCENYYDGQPL
DKNKLPKKA

>BmorCPAP3-A1

MRVFIVLTAVAAIASAQFKCPAKDGQYEDDRQCDKFFECVDGVATTKLCPDGLVFDPTIR
KINKCDQPFNVDCGDRTELQPPKPNSQCPRRNGFFAHPDPSVCNIFYNCIEGEATEVKC
TAGLHFDEYSGTCVWPDSAGRQGCNEQQKKTKDGFECPKEQLVDAQGQIVAHPKFPH
PNDCQRFYVCLNGVEPRDLGCQVGEVY

CPLCP

>BmorCPLCP2

MKTALCLVFLLVAAAAASEKKTTESKAEPLEKKLDKRGLLNLGYGYGIDGLDVGYIGHGQ
GLGGAYNYVDGGYSSGYGLNFGGHTDVTKTITLVKGVPVPYAVDRPVPYPVEKHVPYP
VKVAVPQPYEVVKHVPYHVKEYVKVPVHVPAPYPVEKKVPYPVHVPVDRPYPVKVLVPQ
PYPVEKHVPYPVKVPVPQPYPVEKHVPYPVEVKVPVPQPYPVVKHVGVPVKVPVDRPY
PVHVPAPYPVEKPVPFAVPVEKPVAYPVHVPVDRPYAVHVEKPVAVPVKVPVPQPYPVY
KHVPYAVDRPVAVPVKVPVDRPYPVTVERHVPITVEKPVPVPVKVPYLVSDHHDHHDHH
DHYSQLSYGGSYYGH

>BmorCPLCP1

MRPMLVAASLVALLALAYAEEAKKAEKEVAVTDKEPAADDKKHEKRGLLDIGWHGGFDG
GYGGGGYGGGGYGGGGHYGGHEEVHKTVTVVKKVPVPYPVEKHIPYPVEKKIPYPVK
VHVPQPYPVVKHVPYPVKEIVKVPVHVPQPYPVEKKVPYPVHVPVDRPVPVKVYVPEPY
PVEKKVHVPVEVHVPAPYPVEKKVPYPVKVPVHVPAPYPVYKEVQVPVKVHVDRPYPV
HIPKPVPYPVEKPVPYPVEKPVPYPVKVHVDRPVPVHVEKPVPYPVKVPVPAPYPVEKHI
PYPVEKAVPFPVNIPVDRPYPVHIEKHVPVHIEKPVPYPVKVPVPIVVSHEHGHEHGHDF
GHHGGY

>BmorCPLCP3

MKYTVILVASLAVVAFAKEEKGTPKAVEEKKQDKRGIYDIGSYGGHNFGGSDEGYGGHE
SYGGHEGISFGHQEGHDFGGESYNLGGHGGGEYGSSGGDWKPIASEGDYHHGHHEH
HEHIKTVEVVKKVPVPYTVEKHVPYTVEKKVPYEVKVPVPQPYTVEKKVPFTVKEYVKY
PVYVPEPYTVEKKVPYEVKVHVDKPYEVKVKVPTPYTVEKKIPYEVKVPVPQPYTVEKK
VPVPVKYEVKVPQPYEVIKKVPYEVKVPVDRPYNVYVPKPYPVHVEKPYPVTVHKPVPY
EVKVPVDKPYKVEVEKPYPVPVKVPVPKPYEVIKKIPYTVEKKVPYEVKVPIDKPYPVYK
EVQVPLVKEVPYPVKYHVPIYFKKEEEHHQHHQHHGWH

>BmorCPCFC

MYGKLFAILTLAAVALAREYPAGLHPAICPNYPFCDADALAKYTPQGMPIPEWVRNPAILPI

ARAASNSVPKYPADFPAALCPNYPYCW

>BmorCPFL3

MAAKLVVLLCALATARAGGLYGASYAAPIASYAAAPVIKSYAAPAYAAYSAPAYSAYSAPAY

SAYSAPAYSAYAAPAYSAYAPASYSTYAASPVYKSYSAPAVAVSAPIVKAVAPAVSSVSSYS

TQTSHGAPLLTKTIAPVATYAAAAPVAAYAAHAAPLATYAAHAAPVAYSAPYTTYAHAAPL

VTKTYAAPVASYAHAAPLTYAAAPVLKSAISYSAAPAVSHVSYSGLSGHYGW

>BmorCPT3

MKAFLVLAAVAALGSARPEAGYTYNAPGGGSLGGHGGGIGGGIGGIGGGIGGGHGGFS

SGGLSSSSFGGGLGGGLGGSLGGGFSSGGFSSSGGSFSSGGAIGGGFSGGAIGGGF

GGGFGGAPIVQKHIYVHVPPPEPEEQRPQVISGGAIPQKHYKIIFIKAPAPPAPVAPIIPAQ

AQNEEKTLVYVLVKKPDEQPDITIPTAAPTQPSKPEVYFIKYKTQKEGGSIGGGAIGGGIG

GGLGGIGGGIGGGSIGGGSLGGGISGGSIGGGISGGSLGGGISGGSIGGGHGGSIGGGI

SGGHGGSGVSTSYGPPGHSGPY

>BmorCPF

MILKIVLLCGAVAAVHGGGLIAPAYSAPYSYGAWNPYSSYPAQPALASQHSNTSPFNLGQ
ISTYSKSVDTPFSSVRKADIRVSNPGVAVAPAYSSFAAPYVSHVGVAAPVAKVATTGGLLG
VAYSAAPTVSHMTYTNGLGFAYGW

Supplementary Fig. 2 The detected peptides of CPs from scales. Peptides detected in soluble fraction are highlighted: yellow, only in P-6 scale; grey, only in P-8 scales; turquoise, in P-6 and P-8.The trypsin cleavage site is in red; histidine residues are in blue; signal peptide is underlined.

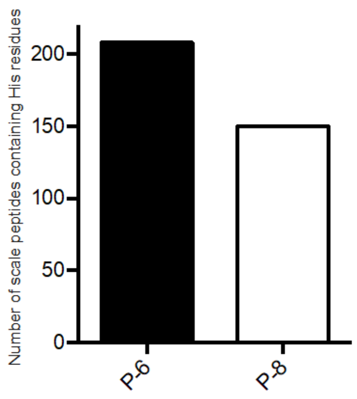

Supplementary Fig. 3 Number of peptides containing His-residues in scales. More peptides containning Histidine were identified in the wing scales of P-6 compared to P-8.

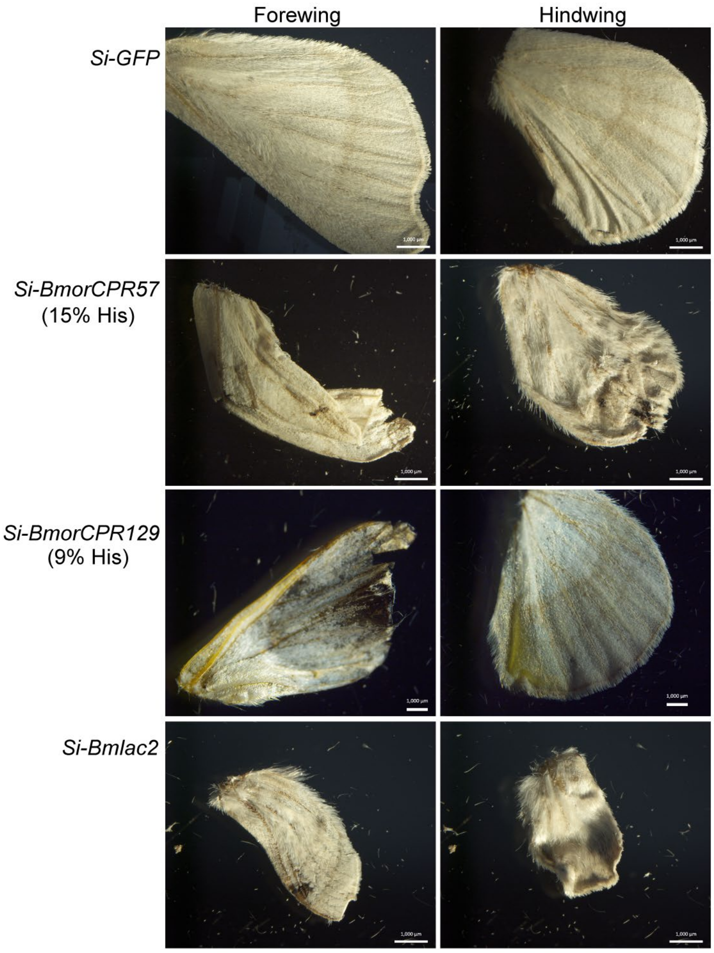

Supplementary Fig. 4 Effect of RNAi with *BmCPs* and *Bmlac2A* on wing development. Wings taken from animals 2 days after eclosion showing that administration of RNAi on P-4 and P-6 directed against transcripts from His-rich genes *Bmlac2A, BmorCPR57 and BmorCPR129* resulted in abnormal wing development. *si-GFP*, green fluorescent protein (control).

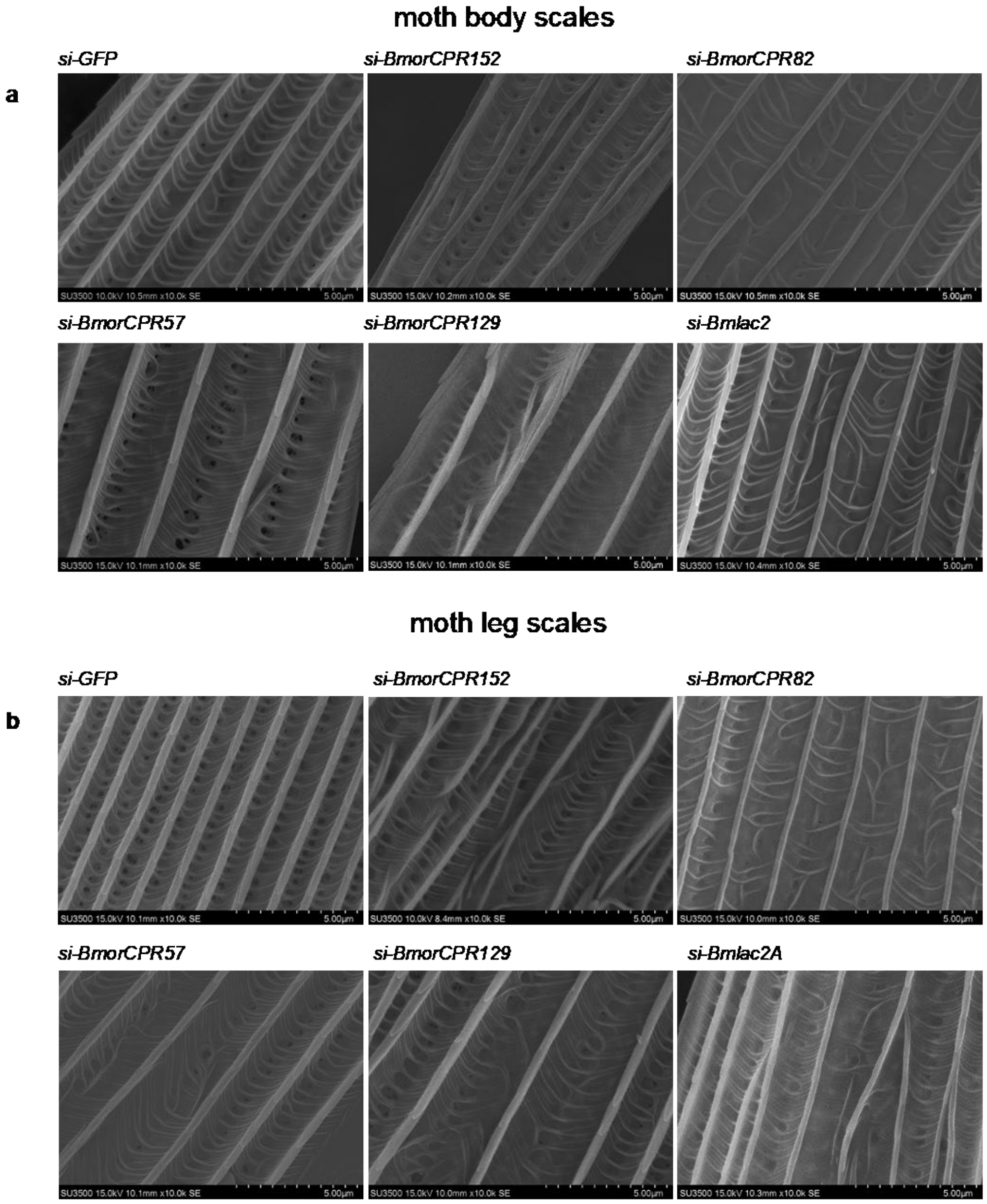

Supplementary Fig. 5 Scale microstructure after injection of siRNA directed against His-rich *CPs* and *Bmlac2A*. The depletion of the mRNA of His-rich *CP* genes *BmorCPR152* (45% His), *BmorCPR82* (27% His), *BmorCPR57* (15% His) or *BmorCPR129* (10% His) caused abnormalities in body scales (a) and leg scales (b) similar to the abnormal wing scales. *si-GPF*, control.

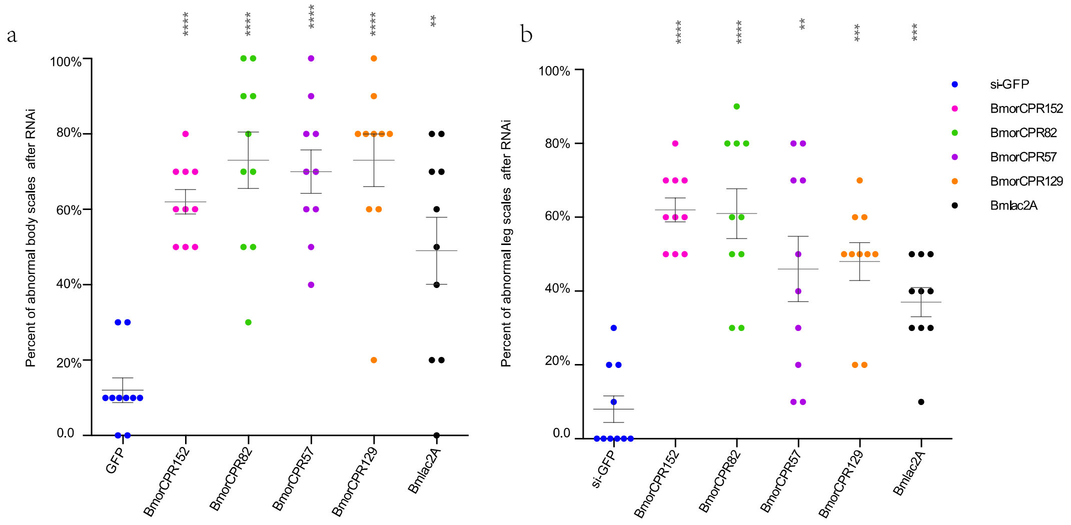

Supplementary Fig. 6 Percent of abnormal scales after interference in the expression of His-rich *CPs* and *Bmlac2A* using RNAi. (a). Percent of abnormal body scales after RNAi treatment. (b). Percent of abnormal leg scales after RNAi treatment (n=9). Significance: ** *p*<0.01, *** *p*<0.001 and ****P<0.0001 (n=10). *si-GFP*, control.

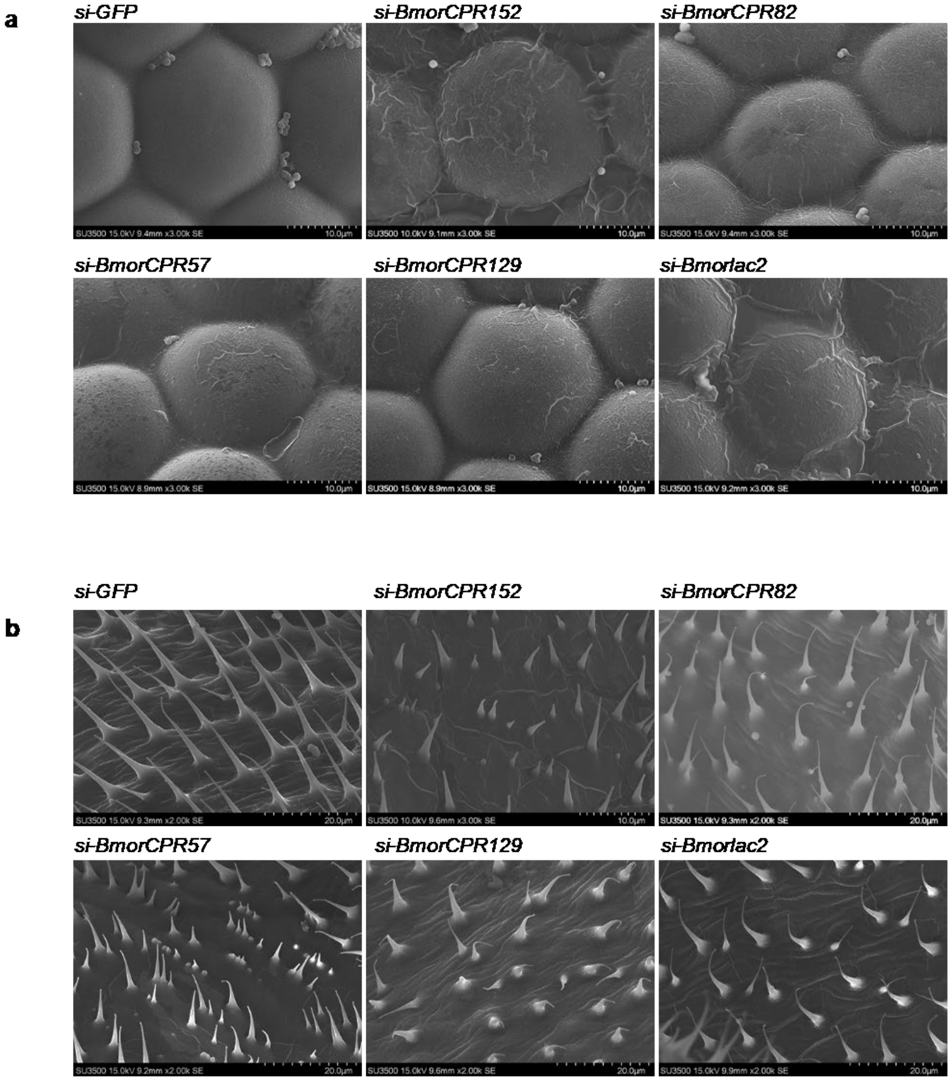

Supplementary Fig. 7 Effect of RNAi treatment with *BmCPs* and *Bmlac2A* on non-wing body parts. Severe damage in the compound eyes (a) and mandibular bristles (b) after interference in the expression of His-rich *CPs* and *Bmlac2A*. *si-GFP*, control.

>BmorCPR152
HPPATSHQFRVDHHDHHDHHHHVDHHNNHHHHVDHDHHSHHDHHRHFN
HNDHHDHHDHHEHQAYHGHHGHDGHHDHHGHHGHHDHHVTHQQHGHL
DHASSYPSYQFSYSVDDHHTGDHKSQSETRHGDHVTGEYSLVEPDGNVR
SVHYNADDHHGFNAVVHNRFAHHHIIPHHDHHHHHDHHNHHDHHEHHDH
HDHHVHHDHHDHHDHHDHGHHEHHSHHDGHHEHHHDHHH

Supplementary Fig. 8 The amino acid sequence of BmorCPR152. XH/XHH/XHHHH repeats covering the His-rich region of BmorCPR152. Blue: His-rich region; magenta, chitin- binding domain (based on Rebers and Willis 2001[1])

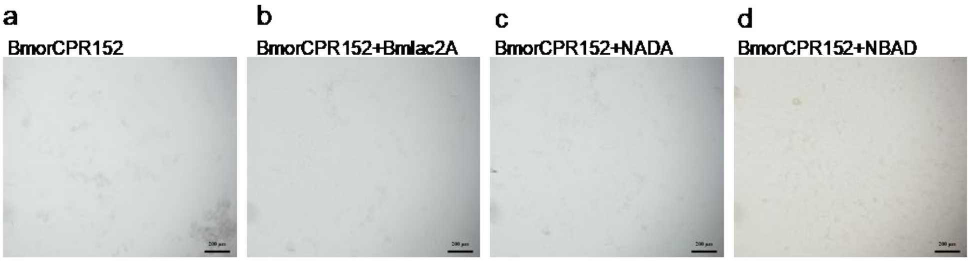

Supplementary Fig. 9 Failure of film synthesis in the absence of Bmlac2A and catechol. (a) Reaction buffer for crosslinking without Bmlac2A and catechol (NADA / NBAD). (b) Reaction system containing BmorCPR152 and Bmlac2A. (c) Reaction system containing BmorCPR152 and NADA. (d) Reaction system containing BmorCPR152 and NBAD. Scale bar =200μm.

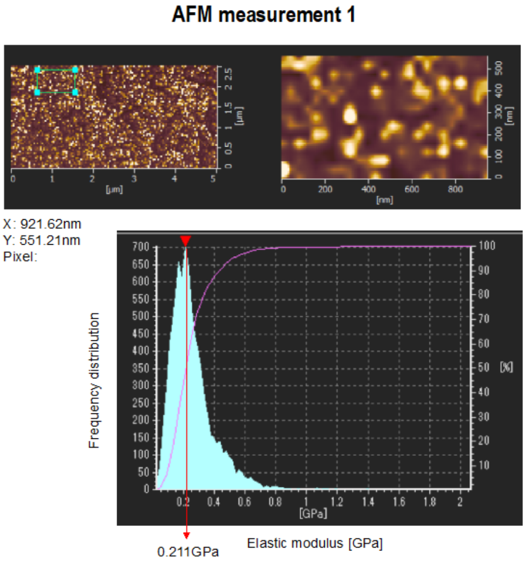

Supplementary Fig. 10 Duplicate AFM measurement of elastic modulus values of films produced with BmorCPR152. The values shown are estimated to be 0.17-0.21 Gpa.

>BmorCPR152

MWFQVATLCLVVIAAMAHPPATSHQFRVDHHDHHDHHHHVDHHNNHH
HHVDHDHHSHHDHHRHFNHNDHHDHHDHHEHQAYHGHHGHDGHHDHH
GHHGHHDHHVTHQQHGHLDHASSYPSYQFSYSVDDHHTGDHKSQSET
RHGDHVTGEYSLVEPDGNVRSVHYNADDHHGFNAVVHNRFAHHHIIP
HHDHHHHHDHHNHHDHHEHHDHHDHHVHHDHHDHHDHHDHGHHEHHS
HHDGHHEHHHDHHH

>BmorCPR82

MYTTVTALFVLVSACQAIFPFHHHHPAISHQEIEKHVGPHHTVGIHH
PVPIPIHHHVPLVHHIPHHHIPIHHDHYAFPEYKFAYSVHDHHTGDV
KSQHELRHGDVVRGGYELVEPDGRFRKVEYKADDHTGFNAIVHHSSP
HHHFHQEHHHI

>BmorCPR57

MFQYIAIACLLGVAAAAPSATPTAAAYVAAVDSVSVPHYGFNYAVNDPHTG
DNKAQTESRDGDVVKGSYSLTEPDGTIRVVDYTADSVTGFNANVKRLGPAA
HPQTLITKQAIVAPVVTHAVASVAHVPTSLISVGIGHGHGGHHYAGLGHIG
HGHGGHDYAGLGHIGHYGIGHVGHADLAHIGHGHIGHLGLGHVDLGYAGIG
HHAHVGLDHIGHIGLGHGGLDHIGHIGLSHGGLDHISHIGLSHAGLDHIGH
GLSLKH

Supplementary Fig. 11 Peptides selected for antibody production. Red, selected peptide.

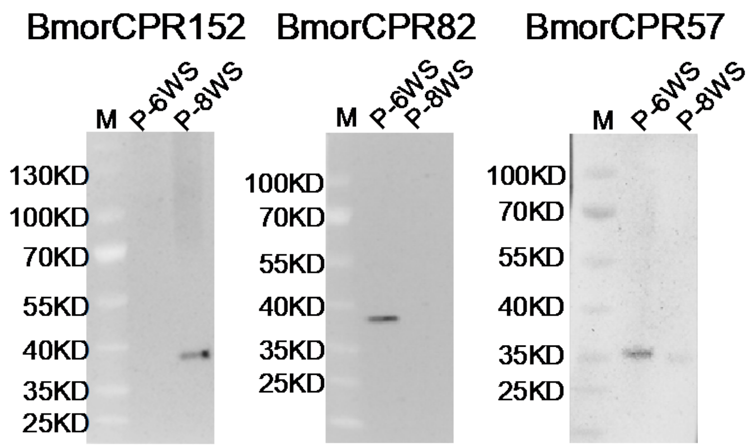

Supplementary Fig. 12 Specificity of antibodies to BmorCPRs examined by Western blot. P-6, proteins extracted from scales of the sixth day after pupation; P-8, proteins extracted from scales of the eighth day after pupation. M, molecular size markers; KD, kilodaltons.

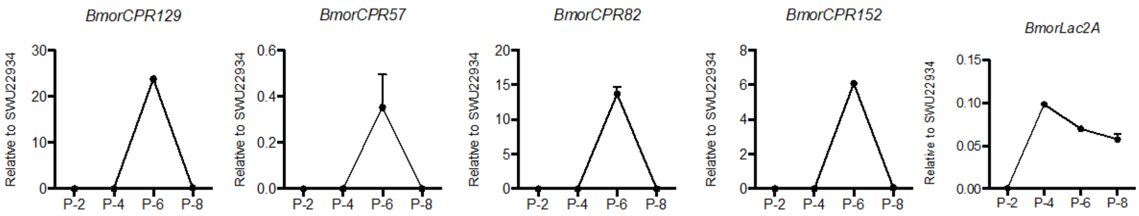

Supplementary Fig. 13 Transcript levels for His-rich *CP* genes and *Bmlac2A* in wings. Transcription was measured relative to levels of *SWU22934* (*B. mori* translation initiation factor 4A) in the pupal stage by qRT-PCR.

Reference

1. Rebers, J. E , Wilis, J. H. A conserved domain in arthropod cuticular proteins binds chitin. *Insect Biochem Mol Biol* **31**, 1083-1093 (2001).
