# Supplementary material for: Lepidopteran wing scales contain abundant cross-linked film-forming histidine-rich cuticular proteins
Source: Commun Biol. 2021 Apr 22;4:491. doi: 10.1038/s42003-021-01996-4 (PMC8062583; doi:10.1038/s42003-021-01996-4)
Supplement: Supplementary file 3 — Description of Additional Supplementary Files [file 42003_2021_1996_MOESM3_ESM.pdf]

Description of Additional Supplementary Files

File name: Supplementary Data 1

Description: Proteins identified from P6-1 wing scales listed by iBAQ.

File name: Supplementary Data 2

Description: Proteins identified from P6-2 wing scales listed by iBAQ.

File name: Supplementary Data 3

Description: Proteins identified from P8-1 wing scales listed by iBAQ.

File name: Supplementary Data 4

Description: Proteins identified from P68-2 wing scales listed by iBAQ.

File name: Supplementary Data 5

Description: Source data of Fig. 2.

File name: Supplementary Data 6

Description: Source data of Supplementary Fig. 6.
