# Supplementary material for: Lepidopteran wing scales contain abundant cross-linked film-forming histidine-rich cuticular proteins
Source: Commun Biol. 2021 Apr 22;4:491. doi: 10.1038/s42003-021-01996-4 (PMC8062583; doi:10.1038/s42003-021-01996-4)
Supplement: Supplementary file 10 — Reporting Summary [file 42003_2021_1996_MOESM10_ESM.pdf]

# nature research

Corresponding author(s): Kazuei Mita

Last updated by author(s): Mar 31, 2021

# Reporting Summary

Nature Research wishes to improve the reproducibility of the work that we publish. This form provides structure for consistency and transparency in reporting. For further information on Nature Research policies, see our Editorial Policies and the Editorial Policy Checklist.

## Statistics

For all statistical analyses, confirm that the following items are present in the figure legend, table legend, main text, or Methods section.

| n/a | Confirmed | |
|---|---|---|
| ☐ | ☒ | The exact sample size (*n*) for each experimental group/condition, given as a discrete number and unit of measurement |
| ☐ | ☒ | A statement on whether measurements were taken from distinct samples or whether the same sample was measured repeatedly |
| ☐ | ☒ | The statistical test(s) used AND whether they are one- or two-sided<br>*Only common tests should be described solely by name; describe more complex techniques in the Methods section.* |
| ☒ | ☐ | A description of all covariates tested |
| ☐ | ☒ | A description of any assumptions or corrections, such as tests of normality and adjustment for multiple comparisons |
| ☒ | ☐ | A full description of the statistical parameters including central tendency (e.g. means) or other basic estimates (e.g. regression coefficient) AND variation (e.g. standard deviation) or associated estimates of uncertainty (e.g. confidence intervals) |
| ☐ | ☒ | For null hypothesis testing, the test statistic (e.g. *F*, *t*, *r*) with confidence intervals, effect sizes, degrees of freedom and *P* value noted<br>*Give P values as exact values whenever suitable.* |
| ☒ | ☐ | For Bayesian analysis, information on the choice of priors and Markov chain Monte Carlo settings |
| ☒ | ☐ | For hierarchical and complex designs, identification of the appropriate level for tests and full reporting of outcomes |
| ☒ | ☐ | Estimates of effect sizes (e.g. Cohen's *d*, Pearson's *r*), indicating how they were calculated |

*Our web collection on statistics for biologists contains articles on many of the points above.*

## Software and code

Policy information about availability of computer code

Data collection: KAIKObase (ver 3.2.2) was used for gene and protein information.

Data analysis: Mascot 2.2 software was used for LC-MS/MS DATA ANALYSIS. Pfam 32.0 was used for protein domain search. Signal peptides of CPs were predicted by SignalP 5.0. siRNA and primer designs were performed by GENETYX-MAC version 16.0.7.

For manuscripts utilizing custom algorithms or software that are central to the research but not yet described in published literature, software must be made available to editors and reviewers. We strongly encourage code deposition in a community repository (e.g. GitHub). See the Nature Research guidelines for submitting code & software for further information.

## Data

Policy information about availability of data

All manuscripts must include a data availability statement. This statement should provide the following information, where applicable:

- Accession codes, unique identifiers, or web links for publicly available datasets
- A list of figures that have associated raw data
- A description of any restrictions on data availability

The LC-MS/MS analysis raw data are available via ProteomeXchange with dataset identifier PXD024401, PXD024365, PXD024362 and PXD02434. Protein lists identified from LC-MS of P6-1/P6-2/P8-1/P8-2 for Table 1 are presented in Supplementary Data 1-4. Data of Fig. 2 were described in Supplementary Data 5; Data of Supplementary Fig. 6 were in Supplementary Data 6. All relevant data are available from corresponding author upon request (to mitakazuei@gmail.com).

# Field-specific reporting

Please select the one below that is the best fit for your research. If you are not sure, read the appropriate sections before making your selection.

☒ Life sciences ☐ Behavioural & social sciences ☐ Ecological, evolutionary & environmental sciences

For a reference copy of the document with all sections, see nature.com/documents/nr-reporting-summary-flat.pdf

# Life sciences study design

All studies must disclose on these points even when the disclosure is negative.

| | |
|---|---|
| Sample size | We used 3μg of proteins extracted from scales for LC-MS/MS analysis to obtain reliable peptide data. The criteria of reliable protein data by LC-MS/MS was described in Section 2.4 in Materials and Methods. |
| Data exclusions | For LC-MS/MS raw data analysis, false discovery rate less than 0.01 (We described in Section 2.4.) |
| Replication | For LC-MS/MS, we repeat twice, while other experiments such as crosslinking, RNAi, qPCR and SEM observation were performed at least more than 3 times. This situation was written in each experiment. |
| Randomization | All samples were randomly taken. |
| Blinding | Concerning scale observation by SEM, we dissected all wings from moths of a group, then SEM observation was carried out for randomly chosen wings and scales. |

# Reporting for specific materials, systems and methods

We require information from authors about some types of materials, experimental systems and methods used in many studies. Here, indicate whether each material, system or method listed is relevant to your study. If you are not sure if a list item applies to your research, read the appropriate section before selecting a response.

## Materials & experimental systems

| n/a | Involved in the study | |
|---|---|---|
| ☐ | ☒ | Antibodies |
| ☒ | ☐ | Eukaryotic cell lines |
| ☒ | ☐ | Palaeontology and archaeology |
| ☐ | ☒ | Animals and other organisms |
| ☒ | ☐ | Human research participants |
| ☒ | ☐ | Clinical data |
| ☒ | ☐ | Dual use research of concern |

## Methods

| n/a | Involved in the study | |
|---|---|---|
| ☒ | ☐ | ChIP-seq |
| ☒ | ☐ | Flow cytometry |
| ☒ | ☐ | MRI-based neuroimaging |

## Antibodies

| | |
|---|---|
| Antibodies used | Peptide synthesis and polyclonal antibody production in rabbits were carried out by Zoonbio Biotechnology Co. (Nanjing, China) and Wuhan Genecreate Biological Engineering Co. (China). Design of antigenic peptides were listed up at Supplementary Fig. 2. |
| Validation | The specificity of each antibody was conformed by Western-blotting (Supplementary Fig. 12). |

## Animals and other organisms

Policy information about studies involving animals; ARRIVE guidelines recommended for reporting animal research

| | |
|---|---|
| Laboratory animals | Bombyx mori strain Dazao, sex: mixed, stage: pupal and adult |
| Wild animals | No. |
| Field-collected samples | No. |
| Ethics oversight | For silkworm, no regulation exist |

Note that full information on the approval of the study protocol must also be provided in the manuscript.
